# Anthropometric Characteristics, Physical Fitness and Motor Coordination of 9 to 11 Year Old Children Participating in a Wide Range of Sports

**DOI:** 10.1371/journal.pone.0126282

**Published:** 2015-05-15

**Authors:** Katrijn Opstoel, Johan Pion, Marije Elferink-Gemser, Esther Hartman, Bas Willemse, Renaat Philippaerts, Chris Visscher, Matthieu Lenoir

**Affiliations:** 1 Institute of Sport and Exercise Studies, Faculty of Health and Social Studies, University of Applied Sciences Nijmegen, Nijmegen, The Netherlands; 2 Center for Human Movement Sciences, University Medical Center Groningen, University of Groningen, Groningen, The Netherlands; 3 Department of Movement and Sports Sciences, Ghent University, Ghent, Belgium; Vanderbilt University, UNITED STATES

## Abstract

**Objectives:**

The aim of this study was to investigate to what extent 9 to 11 year old children participating in a specific sport already exhibit a specific anthropometric, physical fitness and motor coordination profile, in line with the requirements of that particular sport. In addition, the profiles in children with a different training volume were compared and possible differences in training hours per week between children from a low, moderate, and high level of physical fitness and motor coordination were investigated.

**Methods and Results:**

Data of 620 children, 347 boys and 273 girls, who participated in the Flemish Sports Compass were used. Only the primary sport of each child was considered and six groups of sports (Ball sports, Dance, Gymnastics, Martial arts, Racquet sports and Swimming) were formed based on common characteristics. Measurements consisted of 17 tests. Independent T-tests and Mann-Whitney U-tests revealed few differences between the groups of sports and the discriminant analyses with the moderate and low active group did not show any significant results (p > .05). However, when discriminating among the high active children, a 85.2 % correct classification between six groups of sports was found (Wilks’ Λ = .137 and p < .001). Finally, children performing under average on the tests spent significantly fewer hours in sport per week (2.50 ± 1.84 hours) compared to the children performing best (3.25 ± 2.60 hours) (p = .016) and the children performing above average (2.90 ± 1.96 hours) (p = .029) on physical fitness and motor coordination.

**Discussion:**

The study showed that in general, children at a young age do not exhibit sport-specific characteristics, except in children with a high training volume. It is possible that on the one hand, children have not spent enough time yet in their sport to develop sport-specific qualities. On the other hand, it could be possible that they do not take individual qualities into account when choosing a sport.

## Introduction

The benefits of sports participation on physical and mental health are widely recognized [1–5]. Sports participation not only positively influences anthropometric measures like body weight and body composition [6], children’s health also improves in terms of physical fitness [5, 7, 8] which can be considered one of the most important markers of health [5]. In addition, sports participation at a young age positively contributes to the development of the child’s motor coordination since involvement in physical activity provides more opportunities to learn and refine motor skill executions [7, 9]. In children who are actively involved in sports, differences in levels of physical fitness and motor coordination can partly be explained by the amount of hours spent within the sport. For example, Fransen and colleagues [10] found a positive effect of the amount of training hours per week on flexibility (sit and reach), explosive leg power (standing broad jump) and motor coordination (Körperkoordinationstest für Kinder) in 10 to 12 year old boys.

In addition to the positive influence on the child’s general physical profile, involvement in sport is also associated with the development of sport-specific characteristics. The well-documented comparison between adolescent athletes from different types of sports makes it clear that each sport is, to some extent, unique in terms of physical prerequisites, e.g., [11–17]. For example, soccer players demonstrate high levels of both upper and lower body strength for sport-specific actions including throwing-in and kicking the ball [15], while height is the key ingredient to make it to the top in volleyball [12], and motor coordination appears to be crucial in gymnastics [17]. These sport-specific characteristics make it possible to discriminate between athletes of different sports. A discriminant analysis of anthropometric variables and physical fitness characteristics among adolescent female figure skaters, swimmers, volleyball players and tennis players, showed that figure skaters can be discriminated from the other athletes based upon their lower body mass and height, fewer push-ups and lower maximal girth of the biceps [18]. Similarly, Pion and colleagues [19] studied the discriminative power of 22 anthropometric, physical fitness and motor coordination measurements and reported a 96.4% correct classification for 141 adolescent Flemish boys into nine different sports. In sum, the unique characteristics of elite adolescent athletes from different sports have been widely demonstrated, thereby providing important information from the viewpoint of talent detection, identification, and development. However, most of these studies have focused on adolescent and adult athletes that have already benefitted from a considerable training history that has at least in part shaped their current anthropometric, physical fitness and motor coordination profile. The question remains to what degree these specific characteristics are already present in children with a limited training history.

Consequently, the central question in this paper is to what extent young children participating in a specific sport already exhibit a specific anthropometric, physical fitness and motor coordination profile in line with the requirements of that particular sport. This is a relevant question from the perspective of health-enhancing physical activity, as well as from the viewpoint of talent identification. A match between the sport-specific characteristics and the individual anthropometric, physical fitness and motor profile of a child is more than likely an efficient protection from early drop-out from sports participation because the child will experience early success in this sport [20]. Children experiencing early success in a particular sport, not necessarily at a (high) competitive level, might increase their chances for sustained sports participation and an active lifestyle later on [21]. With respect to talent identification, children with a profile that matches the requirements of a specific sport from a young age on will more likely continue training and by consequence have better chances on an optimal talent development pathway.

The first aim of the present study was to examine whether 9 to 11 year old children already involved in sports participation demonstrate sport-specific characteristics in terms of anthropometry, physical fitness and motor coordination. The authors expect that sport-specific profiles are generally not distinctive enough at a young age.

Since training inevitably shapes the individual profiles, the second purpose is to construct sport profiles based on 17 performance measurements and to compare them in children with a low, moderate, and high training volume. Ericsson’s theory of deliberate practice [22] states that the level of expertise obtained by elite athletes is at least in part a function of the amount of structured practice. It was expected that children with a more extended training history would exhibit more pronounced anthropometric, physical fitness and motor coordination profiles matching the specific sport.

In the third aim, we investigated the difference in training hours per week between children from a low, moderate, and high level of physical fitness and motor coordination. Since sports participation contributes positively to the child’s general physical fitness and motor profile, it was expected that children performing better on physical fitness and motor coordination, spend more hours per week in their sport.

## Materials and Methods

### Ethics statement

The Ethics Committee of the Ghent University Hospital approved the study and written informed parental consent was obtained for all participants [23].

### Participants

The data for this study is part of the Flemish Sports Compass (FSC), a cooperation of the Flemish government and Ghent University that was started in 2007 and ended in 2012 [10, 19, 23–25]. Twenty-six primary schools were randomly selected from the five Flemish provinces of the Flemish region and the Brussels-capital region (for details see [23]). A sample of 620 children (10.30 ± 0.88 years), 347 boys and 273 girls, who participated in the FSC and who were involved in at least one sport, were included in the present study. A total of 343 children practiced one sport, 181 children were involved in two different sports and 96 children practiced three different sports. Within this study, the primary sport, i.e. the sport in which the child spends most of the time, was selected, which resulted in a total of 25 different sports.

#### Groups of sports

The 25 different sports were initially divided into 8 sport groups based on common characteristics (see [19]). Recreational running (n = 12) and track and field (n = 23) were placed under ‘Athletics’. Basketball (n = 17), korfball (n = 3), soccer (n = 163) and volleyball (n = 10) were combined as ‘Ball sports’ based on the common character of ball skills. The different types of dancing including ballet (n = 19), folk dance (n = 6), jazz dance (n = 13), modern dance (n = 19) and other dance (n = 54) were combined into the category ‘Dance’. Acrobatics (n = 11), acro gymnastics (n = 6) and artistic gymnastics (n = 38) formed the category ‘Gymnastics’. Judo (n = 21), karate (n = 25) and tae kwon do (n = 5) were combined into the category ‘Martial arts’ and badminton (n = 4) and tennis (n = 42) were both considered ‘Racquet sports’. The rest of the sports did not fit into any of the aforementioned categories: recreational bicycling (n = 15); figure/ice skating (n = 4); field hockey (n = 8); horse riding (n = 35); skiing (n = 6) and swimming (n = 61). Therefore these sports were combined into the category ‘Other sports’ except for swimming. Based on the amount of swimmers (n = 61) and the distinct profile of the sport, swimming was considered as a category of its own. The groups ‘Athletics’ and ‘Other Sports’ were only considered for the descriptive part of this study and not included for other analyses based on the diversity of sport-specific skills within the group.

### Measurements

A subset of 17 tests of the FSC was used in the present study. Trained examiners assessed the children in accordance with the test guidelines of the FSC protocol.

#### Anthropometry

Body height (BH) and sitting height (SH) (0,1 cm) were both measured using portable stadiometers (Harpenden, Holtain Ltd., Crymych, UK). Body weight (BW) (0.1 kg) and body fat percentage (BF) were measured using a bio-electrical impedance device (Tanita, BC-420SMA). Body mass index (BMI) was calculated using the following formula: BMI = (body weight/body height^2^).

#### Physical fitness

Cardiovascular endurance was obtained using the 20-m endurance shuttle run test (SR) (0.5 min) (EUROFIT) [26]. Children had to run back and forth between two lines 20 meters apart, at a speed that was imposed by means of beep signals. As the test progressed, the time provided to reach the other side gradually decreased, requiring the children to run faster and faster. Failure to cross the other line before or on the beep was only allowed once. The SR test has adequate values for validity, ranging from .68 to .76, and reliability, ranging from .68 to .84, measured in 4 to 18 year old children [27].

The sit-and-reach test (SAR) (EUROFIT) [26] was used to assess children’s hamstring and lower back flexibility, with an accuracy of 0.5 cm. The SAR test has adequate validity and reliability values ranging from .60 to .73 and .70 to .98 respectively, measured in 4 to 18 year old children [27]. Shoulder flexibility (SF) (0.5 cm) was assessed using the shoulder rotation test [24, 28, 29]. A lower score indicated better flexibility. The shoulder rotation test proved to be reliable with a test-retest reliability coefficient between .73 and .96, measured in 9 to 13 year old children [28].

The 10x5 shuttle run test (10x5 SR) (EUROFIT) [26] was used to measure the child’s speed and agility. The time children needed to run back and forth as quickly as possible between two lines 5 meters apart, 10 times in a row, reflected their speed and agility. The 10x5 SR test has adequate values for validity, ranging from .62 to .85, and reliability, ranging from .62 to .96, measured in 4 to 18 year old children [27].

This study included four tests to measure children’s strength. Both standing broad jump (SBJ) and counter movement jump (CMJ) measured the child’s explosive leg power with an accuracy of 1.0 cm and 0.1 cm respectively (EUROFIT) [26]. The SBJ showed adequate values for validity and reliability ranging from .52 to .78 and .66 to .97 respectively [27]. The CMJ showed high values for validity and reliability with .87 for internal consistency and a Cronbach’s α of .98 for reliability [30]. The highest of three counter movement jumps, measured by means of an Optojump device (Microgate, Bolzano, Italy) [31], was used for further analysis. Muscular strength and muscular endurance of the upper body were obtained using sit-ups (SU) and knee push-ups (KPU) (BOT-2) [32]. The participants were asked to perform as many repetitions as possible within 30 seconds. The SU and KPU proved to be reliable and valid tests for strength with a test-retest reliability coefficient of .88, measured in 8 to 12 year old children, and an intercorrelation coefficient of .87, measured in 8 to 11 year old children [32].

#### Motor coordination

Gross motor coordination was measured using the Körperkoordinationstest für Kinder (KTK) [33]. Three subtests were included in this study. For balance, children were asked to walk backwards (WB) on three different balance beams with decreasing width. Three attempts on each of the three balance beams resulted in a total score of maximum 72. For the second test, children had to jump sideways (JS) with both feet together over a wooden slat, as fast as possible. The sum of two attempts of 15 seconds resulted in a total score. Finally, for the test moving sideways (MS), children were asked to make as much relocations as possible within 20 seconds by means of two 20 by 20 cm square boxes. The sum of two attempts resulted in a total score. The scores of each of the three subtests were then converted into age- and gender- specific motor quotients [25]. The KTK proved to be a reliable instrument with test-retest reliability coefficients of .80, .95 and .84 for WB, JS and MS respectively.

Upper limb coordination was measured by dribbling a tennis ball (BD) with alternating hands 10 times in a row (Short form Bot-2) [32]. The score equals the number of correct dribbles with a maximum of 10. When the child did not reach the maximum score of 10, a second trial was conducted. The upper-limb coordination subtest showed adequate values for reliability and validity with a test-retest reliability coefficient of .59, measured in 8 to 12 year old children, and an intercorrelation coefficient of .82, measured in 8 tot 11 year old children [32].

### Sports participation

The Flemish Physical Activity Computerized Questionnaire (FPACQ) [34] was used to obtain the type of organized sport children participated in and the amount of training hours per week at the time of data collection. The primary sport was taken into account for this study. The FPACQ proved to be a reliable and valid instrument to measure the amount of hours of sports participation per week with a test-retest reliability coefficient of .74 and a Pearson correlation coefficient of .52 for concurrent validity [34]. To ascertain the validity, the FPACQ was compared to the output measures of the Computer Science and Applications uniaxial accelerometer.

### Data analysis

Data were analyzed using SPSS version 20.0. Significance level was set at P < .05. Descriptive statistics were obtained for the absolute values of each of the 17 performance measurements for the 25 different sports separately and for the eight groups of sports. To allow the comparison of the results of children from different ages (9, 10 and 11 year old children), standardized Z-scores were calculated using the age specific means for each of the 17 variables.

#### Sport-specific characteristics

To examine whether 9 to 11 year old children already involved in sports participation demonstrate sport-specific characteristics in terms of anthropometry, physical fitness and motor coordination Independent T-tests (in case of normally distributed data) or Mann-Whitney U-tests (in case of not normally distributed data) were performed. The Shapiro-Wilk test was used to test for normality of data. For each of the 17 performance measurements, the Z-score of each of the six groups of sports (Ball sports, Dance, Gymnastics, Martial arts, Racquet sports and Swimming) was compared to an overall Z-score of the remaining groups (e.g., body height of the ball sport players vs. body height of the non-ball sport players).

#### Role of training in sport-specific profiles

Three discriminant analyses were performed to construct and subsequently compare profiles of six different groups of sports in children spending one hour or less per week (low active), children spending between one and five hours per week (moderate active) and children spending five or more hours per week (high active). The profiles are based on the Z-scores of the 17 performance measurements, which were inserted as the independent variables. The six groups of sports were used as grouping variable. Discriminant functions and the amount of correctly classified children were calculated.

#### Role of training in PQ and MQ levels

To examine the possible differences in training hours per week between children from a low, moderate, and high level of physical fitness and motor coordination, a One-way ANOVA (in case of normally distributed data) or a Kruskal-Wallis test and three subsequent Mann-Whitney U-tests (in case of not normally distributed data) were performed. The Shapiro-Wilk test was used to test for normality of data. The following three groups were considered: the under average performers with a physical fitness quotient (PQ) and/or motor quotient (MQ) of .0 or lower, children performing above average with a PQ and/or MQ between .0 and .5, and the best performers with a PQ and MQ of .5 or higher. PQ and MQ were calculated using the Z-scores of each of the physical fitness and motor coordination variables (PQ = Z-SR + Z-SF + Z-SAR + Z-10x5 SR + Z-SBJ + Z-CMJ + Z-SU + Z-KPU and MQ = Z-JS + Z-MS + Z-WB + Z-BD).

## Results

### Descriptive statistics

Table 1 shows the absolute values of the anthropometric measures body height (BH), sitting height (SH), body weight (BW), body fat percentage (BF), and body mass index (BMI) for each of the 25 different sports and the eight groups of sports. Table 2 presents the absolute values of the physical fitness measures endurance shuttle run (SR), shoulder flexibility (SF), sit-and-reach (SAR), 10x5 shuttle run (10x5 SR), standing broad jump (SBJ), counter movement jump (CMJ), sit-ups (SU) and knee push-ups (KPU) for each of the 25 different sports and the eight groups of sports. Table 3 displays the absolute values of the motor coordination measures jumping sideways (JS), moving sideways (MS), walking backwards (WB) and ball dribbling (BD) for each of the 25 different sports and the eight groups of sports.

**Table 1:** Descriptive statistics (mean and standard deviation) for the anthropometric variables.

	n	Body height (cm)	Sitting height (cm)	Body weight (kg)	Body fat (%)	BMI (kg/m^2^)
**Athletics**	35	141,03±10,27	73,58±4,66	35,12±10,14	18,45±5,99	17,35±2,75
Recreational running	12	139,30±11,59	73,19±5,23	37,38±12,23	20,15±7,24	18,78±2,99
Track and field	23	141,93±9,67	73,78±4,45	33,93±8,93	17,57±5,18	16,61±2,36
**Ball sports**	193	141,65±7,21	74,26±3,55	34,90±6,98	17,24±6,00	17,29±2,55
Basketball	17	143,60±7,60	75,04±3,24	38,04±6,61	19,55±6,62	18,39±2,28
Korfball	3	142,53±7,43	75,10±3,73	35,43±2,25	16,03±2,46	17,46±0,80
Soccer	163	141,36±6,92	74,14±3,50	34,33±6,71	16,71±5,80	17,09±2,54
Volleyball	10	142,71±10,97	74,63±4,90	38,65±10,53	22,18±6,40	18,66±3,00
**Dance**	111	142,38±7,54	74,44±3,93	34,83±7,47	18,43±6,27	17,03±2,47
Ballet	19	145,42±8,10	75,38±3,62	35,62±7,34	17,58±5,52	16,68±1,94
Folk dance	6	143,72±9,90	75,03±5,50	34,87±7,76	18,52±8,14	16,85±3,40
Jazz dance	13	139,45±7,67	73,30±3,31	34,69±8,48	20,37±6,34	17,62±2,71
Modern dance	19	141,57±7,69	74,45±5,08	32,63±6,81	17,38±6,44	16,13±2,21
Other dance	54	142,15±6,88	74,32±3,58	35,36±7,59	18,62±6,34	17,34±2,53
**Gymnastics**	55	141,37±8,10	73,67±3,89	34,13±7,00	17,56±6,23	16,94±2,38
Acrobatics	11	143,07±7,97	74,76±3,29	37,35±7,92	19,89±7,80	18,12±3,05
Acro gymnastics	6	137,90±5,21	71,63±3,08	31,28±3,45	18,37±2,11	16,42±1,07
Artistic gymnastics	38	141,43±8,50	73,67±4,10	33,64±6,95	16,76±6,11	16,68±2,26
**Martial arts**	51	142,87±8,44	74,68±3,84	35,89±7,25	18,56±5,65	17,45±2,26
Judo	21	142,10±7,94	74,05±4,12	34,49±7,10	17,89±6,09	16,93±2,20
Karate	25	143,13±9,10	75,28±3,86	36,28±6,59	18,37±5,15	17,62±2,12
Tae kwon do	5	144,76±8,32	74,36±2,40	39,82±10,69	22,34±5,80	18,73±3,06
**Other sports**	68	141,25±6,97	74,13±3,86	34,85±7,50	19,33±7,83	17,37±3,02
Bicycling (recreational)	15	142,25±6,18	74,55±2,99	40,09±9,74	23,17±10,78	19,67±4,01
Figure/Ice skating	4	143,53±12,76	74,18±6,55	35,73±5,57	20,08±2,35	17,31±1,45
Field hockey	8	140,29±6,81	73,51±2,75	33,31±5,59	17,65±8,56	16,97±3,11
Horse-riding	35	140,83±7,04	74,07±4,35	32,75±5,77	17,59±5,91	16,44±2,02
Skiing	6	140,95±5,85	74,18±2,93	35,47±8,99	21,67±8,66	17,69±3,44
**Racquet sports**	46	141,61±6,59	73,77±3,10	33,79±5,59	17,99±5,74	16,78±2,02
Badminton	4	143,78±3,54	73,35±0,82	36,18±3,73	19,28±6,30	17,55±2,24
Tennis	42	141,41±6,80	73,81±3,24	33,56±5,71	17,87±5,75	16,71±2,02
**Swimming**	61	141,87±8,29	74,55±4,45	35,56±7,56	19,34±6,42	17,51±2,56

**Table 2:** Descriptive statistics (mean and standard deviation) for the physical fitness variables.

	n	SR (min)	SF (cm)	SAR (cm)	10x5 SR (s)	SBJ (cm)	CMJ (cm)	SU (n/30s)	KPU (n/30s)
**Athletics**	35	5,83±2,22	91,4±15,1	20,1±6,6	22,0±2,0	141,7±20,7	20,4±4,8	22,8±7,0	26,2±6,3
Recreational running	12	5,63±2,30	87,3±20,4	23,1±7,1	21,8±2,2	143,6±21,9	19,3±4,5	20,7±9,1	25,3±7,1
Track and field	23	5,93±2,22	93,5±11,4	18,5±5,9	22,1±1,8	140,7±20,5	20,9±4,9	23,9±5,6	26,6±5,9
**Ball sports**	193	6,05±2,38	91,1±17,5	18,7±5,7	21,9±1,6	141,0±20,5	20,5±4,1	22,6±6,8	26,5±6,4
Basketball	17	5,62±1,89	92,7±20,1	19,6±6,2	22,3±1,5	133,8±15,9	19,4±2,9	22,8±7,4	26,9±6,3
Korfball	3	7,50±1,32	95,0±8,7	17,3±2,1	20,0±1,4	165,3±9,5	25,0±3,3	27,7±0,6	28,0±9,0
Soccer (field)	163	6,11±2,43	90,6±17,4	18,5±5,7	21,9±1,5	141,7±20,6	20,6±4,1	22,6±6,7	26,3±6,4
Volleyball	10	5,35±2,57	96,4±16,6	20,6±5,8	21,9±2,2	135,0±22,4	19,7±3,9	19,8±8,1	27,4±6,5
**Dance**	111	5,35±2,37	88,7±13,7	21,2±5,6	22,0±1,5	140,2±21,1	20,0±4,2	22,9±6,4	25,3±7,0
Ballet	19	5,74±2,40	87,3±11,3	19,8±5,1	21,5±1,4	147,8±21,3	21,8±4,0	23,9±7,2	27,3±5,5
Folk dance	6	3,83±1,21	91,0±11,7	18,9±3,3	22,1±1,3	124,8±16,6	17,9±4,8	20,5±6,9	22,8±7,5
Jazz dance	13	5,04±1,80	86,0±11,5	22,3±5,3	22,1±1,2	141,3±14,7	18,8±3,9	22,8±7,1	25,5±6,9
Modern dance	19	4,11±1,89	92,9±10,4	22,7±5,7	22,6±1,3	131,2±19,9	20,2±4,5	24,7±6,6	21,2±6,0
Other dance	54	5,89±2,53	88,1±15,9	21,1±5,9	21,9±1,8	142,1±21,8	19,9±4,2	22,2±5,7	26,3±7,3
**Gymnastics**	55	5,35±2,18	87,3±16,6	21,1±7,8	21,9±2,1	141,6±24,5	21,5±4,8	24,7±8,5	26,4±7,5
Acrobatics	11	5,09±1,88	93,3±20,9	18,1±10,8	22,3±1,8	134,3±26,0	21,0±5,0	22,5±7,1	28,3±9,8
Acro gymnastics	6	5,75±1,72	71,7±8,2	29,4±6,3	21,8±0,8	156,8±12,8	22,0±3,6	30,0±10,5	23,7±5,2
Artistic gymnastics	38	5,37±2,35	88,1±14,9	20,7±6,1	21,8±2,3	141,3±24,9	21,6±5,0	24,6±8,4	26,3±7,1
**Martial arts**	51	5,12±2,04	91,4±15,9	17,5±6,9	22,5±1,9	138,7±23,9	20,3±4,3	22,0±7,2	26,5±6,6
Judo	21	5,05±1,93	89,6±13,5	19,0±7,0	22,6±,9	139,4±25,1	21,3±4,3	21,7±7,0	25,9±7,7
Karate	25	5,16±2,13	90,9±17,8	17,1±7,1	22,2±1,7	140,0±24,0	19,9±3,8	22,9±7,9	27,0±5,6
Tae kwon do	5	5,20±2,49	101,8±13,8	13,1±3,5	23,5±2,5	129,8±20,3	18,1±5,7	18,6±2,1	26,6±8,0
**Other sports**	68	4,67±2,31	92,2±15,2	19,0±5,9	22,2±1,8	137,7±22,8	19,8±4,5	22,8±6,9	23,8±6,2
Bicycling (recreational)	15	4,40±2,48	88,1±14,0	20,0±5,1	22,3±2,0	132,3±28,6	18,6±5,2	21,7±8,3	23,5±7,6
Figure/Ice skating	4	4,50±1,08	104,0±6,4	21,6±2,7	22,5±1,3	141,3±19,0	19,8±2,9	25,8±5,3	23,0±2,7
Field hockey	8	4,94±2,53	97,6±8,8	18,6±5,6	21,9±1,5	132,4±24,6	18,6±3,1	22,1±6,4	21,4±5,9
Horse-riding	35	4,81±2,45	91,4±15,2	18,2±5,9	22,2±2,0	141,5±21,8	20,5±4,8	22,9±6,7	24,5±6,1
Skiing	6	4,25±1,67	92,0±25,0	20,3±9,8	21,7±0,7	134,0±10,3	19,6±2,0	23,5±7,3	24,8±5,0
**Racquet sports**	46	5,39±1,98	88,9±14,0	17,6±6,3	22,2±1,7	139,8±21,9	20,1±4,1	22,0±6,7	25,4±6,3
Badminton	4	3,88±1,11	90,0±17,8	18,0±9,5	22,0±1,7	141,0±21,6	21,1±4,3	25,3±3,9	25,3±3,9
Tennis	42	5,54±2,00	88,8±13,8	17,6±6,1	22,2±1,7	139,7±22,2	20,0±4,1	21,7±6,8	25,4±6,5
**Swimming**	61	5,27±1,88	90,2±15,3	18,9±7,0	22,6±2,2	138,1±20,5	20,4±4,2	23,7±6,2	26,2±6,9

SR: shuttle run, SF: shoulder flexibility, SAR: sit-and-reach, 10x5SR: 10x5 shuttle run, SBJ: standing broad jump, CMJ: counter movement jump, SU: sit-ups, KPU: knee push-ups

**Table 3:** Descriptive statistics (mean and standard deviation) for the motor coordination variables.

	n	Jumping sideways (n)	Moving sideways (n)	Walking backwards (n)	Ball dribbling (n)
**Athletics**	35	62,9±12,0	43,7±7,4	49,9±12,3	8,77±2,04
Recreational running	12	64,3±11,1	45,9±5,2	49,7±8,9	9,08±1,44
Track and field	23	62,1±12,6	42,6±8,2	50,0±14,0	8,61±2,31
**Ball sports**	193	63,1±11,0	42,6±6,4	45,2±13,2	9,02±2,03
Basketball	17	56,5±12,0	42,4±5,3	39,6±8,0	9,88±0,49
Korfball	3	74,7±9,0	47,3±4,5	60,7±7,1	10,00±0,00
Soccer (field)	163	63,7±10,2	42,7±6,3	45,4±13,0	8,92±2,09
Volleyball	10	60,6±17,8	41,1±8,4	46,1±19,9	8,80±2,57
**Dance**	111	63,5±10,9	43,3±6,9	47,3±13,6	8,39±2,30
Ballet	19	64,4±11,4	42,7±5,6	49,1±8,4	8,42±2,19
Folk dance	6	57,7±13,5	42,0±8,8	42,3±11,0	8,17±2,23
Jazz dance	13	60,9±7,7	40,2±5,1	46,6±13,6	8,00±2,55
Modern dance	19	61,4±9,3	43,2±4,9	43,5±15,9	9,05±1,68
Other dance	54	65,2±11,5	44,4±8,0	48,7±14,4	8,26±2,50
**Gymnastics**	55	62,8±12,5	43,2±7,5	49,2±13,9	8,38±2,55
Acrobatics	11	62,5±14,1	42,8±8,6	48,5±16,7	7,91±2,70
Acro gymnastics	6	61,2±6,0	40,5±6,1	52,7±16,4	8,67±2,07
Artistic gymnastics	38	63,2±13,1	43,8±7,5	48,9±12,9	8,47±2,62
**Martial arts**	51	60,8±12,7	40,8±7,9	43,2±13,5	8,00±2,66
Judo	21	61,1±12,5	40,6±8,9	41,5±11,7	7,48±2,77
Karate	25	62,0±11,7	42,1±5,9	45,1±13,6	8,32±2,51
Tae kwon do	5	53,8±18,7	34,8±10,5	41,0±21,2	8,60±3,13
**Other sports**	68	60,6±13,7	42,0±6,8	47,9±15,7	8,66±2,36
Bicycling (recreational)	15	62,0±18,4	40,3±8,0	46,1±20,5	8,93±1,94
Figure/Ice skating	4	51,0±11,9	43,5±4,8	41,5±24,0	8,00±2,83
Field hockey	8	63,1±12,2	39,3±3,4	45,4±14,4	7,25±3,01
Horse-riding	35	59,7±12,4	42,5±7,2	48,6±13,2	8,94±2,31
Skiing	6	65,2±10,4	45,7±3,8	56,2±12,6	8,67±2,42
**Racquet sports**	46	64,1±13,1	43,2±7,0	46,9±13,6	9,00±1,90
Badminton	4	66,5±16,5	44,5±7,7	42,8±18,0	8,50±3,00
Tennis	42	63,9±12,9	43,1±7,0	47,3±13,4	9,05±1,81
**Swimming**	61	61,7±11,5	41,5±6,1	46,6±14,4	9,02±2,01

### Sport-specific characteristics

The Shapiro-Wilk test pointed out that the variables were not normally distributed (with p-values < 0.05), except for BH (p = 0.690), CMJ (p = 0.120) and MS (p = 0.260). Therefore, the Independent T-test was used for the variables BH, CMJ en MS. The Mann-Whitney U-test was used for the other 14 variables (BW, SH, BMI, BF, SF, SBJ, SAR, 10x5 SR, SU, KPU, SR, JS, WB and BD). The Mann-Whitney U-tests and Independent T-tests revealed that the ball sport players, dancers and swimmers did not show any significant differences from the other children (p > .05). The gymnasts however, performed significantly better on the CMJ (21.51 ± 4.81 cm vs .20.32 ± 4.13 cm) (t(515) = 2.898 and p = .004) compared to the other children. Secondly, in martial arts, children performed significantly lower on the ball dribbling test (BD) (8.00 ± 2.66 correct dribbles vs .8.79 ± 2.16 correct dribbles) (U = 9456.5, Z = -2.412 and p = .016) and scored significantly lower on moving sideways (MS) (40.76 ± 7.858 relocations vs 42.76 ± 6.673 relocations) (t(515) = -2.100 and p = .036) in comparison with the other children. Finally, children involved in racquet sports were significantly less flexible in terms of SAR (17.62 ± 6.31 cm vs .19.46 ± 6.39 cm) (U = 8761, Z = -2.143 and p = .032) compared to the other children.

### Role of training in sport-specific profiles

**Fig 1:**
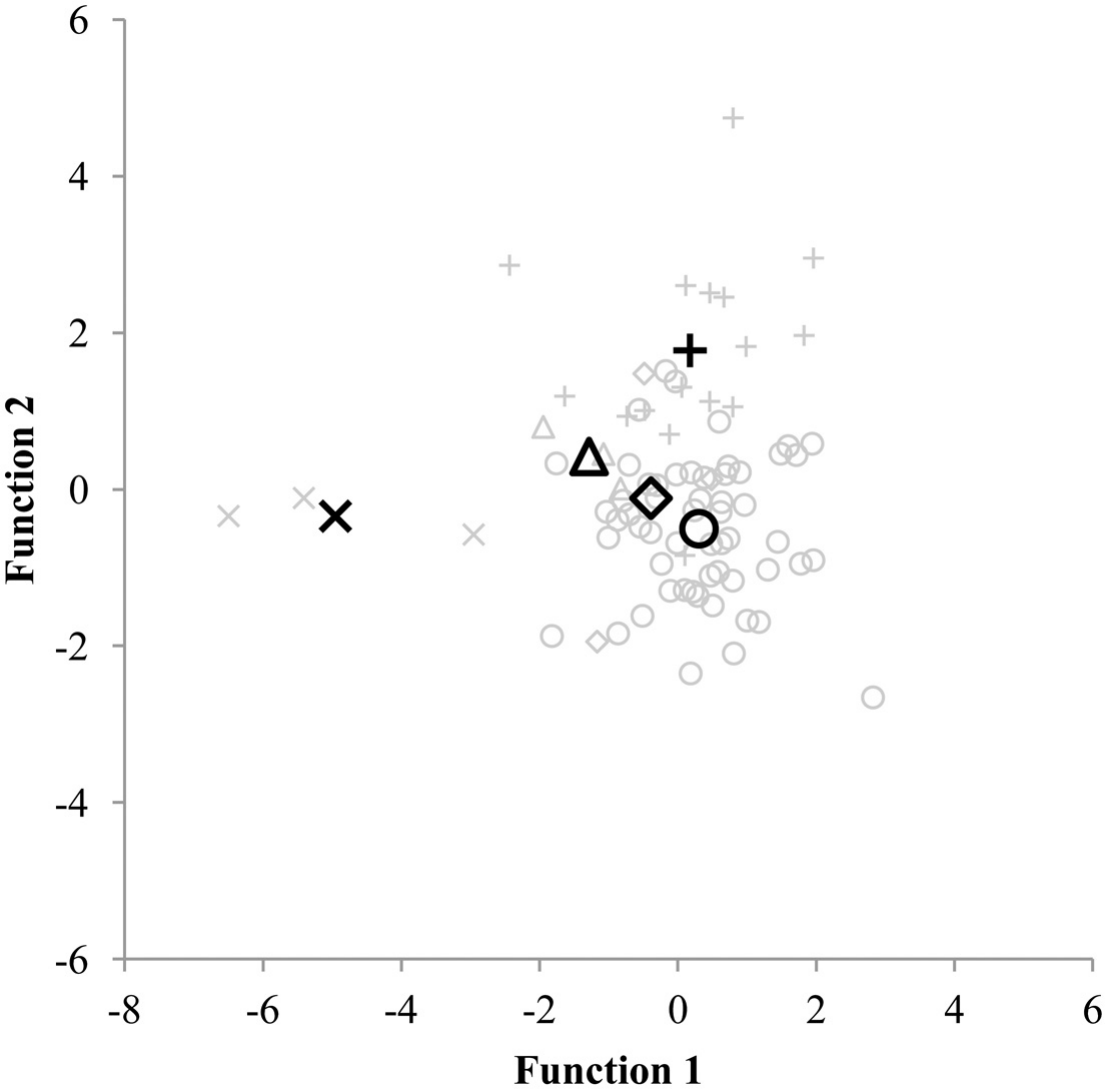
Discriminating between 81 children participating 5 hours or more per week in their sport. Functions at Group Centroids: Ball sports Function 1 = 0.305; Ball sports Function 2 = -0.506; Dance Function 1 = -0.389; Dance Function 2 = -0.114; Gymnastics Function 1 = 0.176: Gymnastics Function 2 = 1.773; Racquet sports Function 1 = -1.285; Racquet sports Function 2 = 0.418; Swimming Function 1 = -4.954; Swimming Function 2 = -0.344. Ball sports = ○; Dance = ◆; Gymnastics = +; Racquet sports = Δ; Swimming = ×.

**Fig 2:**
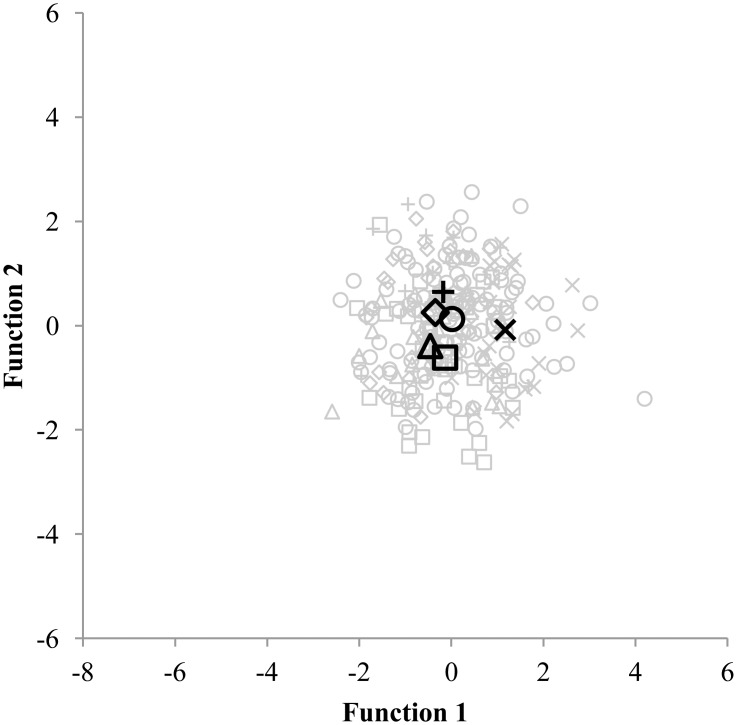
Discriminating between 252 children participating between 1 and 5 hours per week in their sport. Functions at Group Centroids: Ball sports Function 1 = 0.016; Ball sports Function 2 = 0.126; Dance Function 1 = -0.348; Dance Function 2 = 0.250; Gymnastics Function 1 = -0.177: Gymnastics Function 2 = 0.646: Martial arts Function 1 = -0.136; Martial arts Function 2 = -0.615; Racquet sports Function 1 = -0.457; Racquet sports Function 2 = -0.393; Swimming Function 1 = 1.170; Swimming Function 2 = -0.082. Ball sports = ○; Dance = ◆; Gymnastics = +; Martial arts = ☐; Racquet sports = Δ; Swimming = ×.

**Fig 3:**
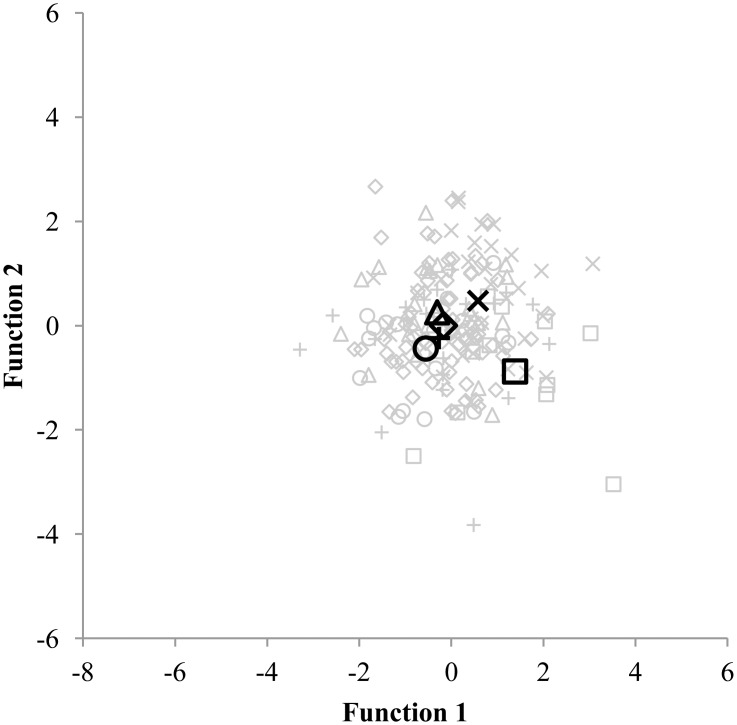
Discriminating between 184 children participating 1 hour or less per week in their sport. Functions at Group Centroids: Ball sports Function 1 = -0.556; Ball sports Function 2 = -0.441; Dance Function 1 = -0.169; Dance Function 2 = 0.000; Gymnastics Function 1 = -0.270: Gymnastics Function 2 = -0.240: Martial arts Function 1 = 1.384; Martial arts Function 2 = -0.881; Racquet sports Function 1 = -0.307; Racquet sports Function 2 = 0.254; Swimming Function 1 = 0.580; Swimming Function 2 = 0.475. Ball sports = ○; Dance = ◆; Gymnastics = +; Martial arts = ☐; Racquet sports = Δ; Swimming = ×.

The first discriminant analysis served to discriminate between 81 highly active children who spent 5 hours or more per week in their sport. Four discriminant functions emerged (Wilks’ Λ = .137 and p < .001) and an 85.2% correct classification was found. Since none of the highly active children were involved in martial arts, only 5 groups of sports (Ball sports, Dance, Gymnastics, Racquet sports and Swimming) were included for this discriminant analysis. For the second and third discriminant analysis, which involved moderate and low active children, all six groups of sports were represented. The second discriminant analysis aimed to discriminate between 252 moderate active children who spend between 1 and 5 hours per week in one of the six groups of sports. Five discriminant functions emerged but were found to be non-significant (Wilks’ Λ = .682 and p = .291). Only 48.8% of the children were correctly classified into their primary sport. Finally, the third discriminant analysis served to discriminate between 184 low active children who spend 1 hour or less per week in one of the six groups of sports. The five discriminant functions that emerged were non-significant (Wilks’ Λ = .577 and p = .230) and 48.4% of the children were correctly classified. The results of the three discriminant analyses are displayed in Figs 1, 2 and 3.

### Role of training in PQ and MQ levels

**Fig 4:**
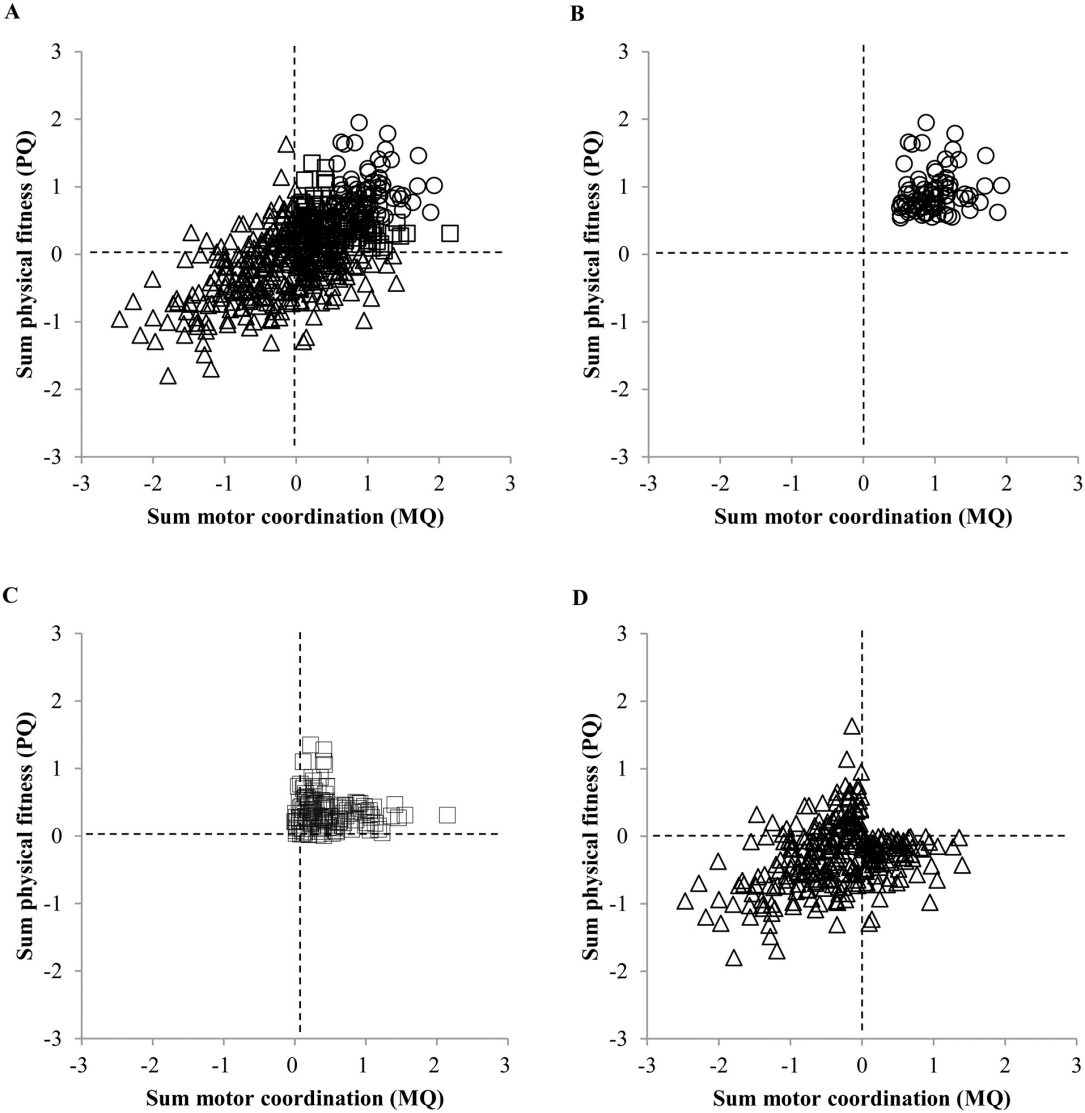
Scatterplot of physical fitness (PQ) and motor coordination (MQ). A. Total sample, B. Children performing best, C. Children performing above average, D. Children performing under average. Best performers = ○ (PQ and MQ > 0.5); Above average performers = ☐ (PQ and MQ > 0 & PQ or MQ < 0.5); Under average performers = Δ (PQ and/or MQ < 0).

The Shapiro-Wilk test showed that the variable ‘amount of hours per week’ was not normally distributed (p < .001). The Kruskal-Wallis test revealed a significant difference in the amount of hours per week spent in the primary sport between the three different groups (χ^2^(2) = 8,315 and p = .016). The children performing under average on PQ and MQ spent significantly fewer hours in sport (2.50 ± 1.84 hours per week) compared to the children performing best (3.25 ± 2.60 hours per week) (U = 9640.5, Z = -2.406 and p = .016) and the children performing above average (2.90 ± 1.96 hours per week) (U = 18597, Z = -2.185 and p = .029). Children scoring best on PQ and MQ did not significantly differ from the ‘above average group’ in terms of hours of sport per week (U = 5699, Z = -.629 and p = .529). In Fig 4a, MQ is plotted against PQ in which the difference is made between the children from a high, moderate and low level of physical fitness and motor coordination. Fig 4b–4d present the MQ/PQ plot for these three levels separately. A positive MQ/PQ equals a score above the average score of the group. Zero represents the average score of the group. A negative MQ/PQ equals a score under the average score of the group. Fig 4b presents the PQ and MQ scores for the children performing best, i.e. a score of .5 or higher on both PQ and MQ. In Fig 4c, PQ and MQ levels are shown for the children performing above average with a PQ and/or MQ between .0 and .5. Finally, Fig 4d presents the PQ and MQ scores of the children performing under average with a PQ and/or MQ of .0 or lower.

## Discussion

The main aim of this study was to examine whether 9 to 11 year old children already involved in sports participation demonstrate sport-specific characteristics in terms of anthropometry, physical fitness and motor coordination. The current study showed that in general, children at a young age do not present sport-specific physical characteristics except in children with a high training volume. Another result is that, regardless of the type of sport, children with the best physical fitness and motor coordination characteristics are the ones who train the most hours per week.

The few differences between the six groups of sports included within this study (Ball sports, Dance, Gymnastics, Martial arts, Racquet sports and Swimming) comprised of the better jumping abilities of the gymnasts, the poorer flexibility of the racquet sport players and the poorer ball skills and the partly poorer gross motor coordination (only in terms of the moving sideways test) of the children involved in martial arts. These differences however, do not entirely correspond with the sport-specific profiles formed by extensive research. Adolescent and adult gymnasts are characterized by their flexibility, strength, coordination, jumping capabilities, anaerobic endurance and distinct anthropometric profile [17, 35, 36]. Within this study, gymnasts only distinguished themselves with better jumping abilities. Literature regarding this topic is inconclusive. Bencke and colleagues [35] found that 11 year old gymnasts showed better jumping capabilities compared to swimmers, handball players and tennis players of the same age. Meanwhile, Pion and colleagues [19] found that male gymnasts with an average age of 16.1 ± 0.8 years displayed poorer jumping capabilities compared to non-gymnasts (including badminton, basketball, handball, judo, soccer, table tennis, triathlon and volleyball). When considering the racquet sport players, it must be noted that the larger part of the group (n = 46) played tennis (n = 42). Therefore, it is likely that the contribution of the badminton players was rather small. With this in mind, we could state that within this study, the tennis players are less flexible compared to the rest of the children, which however, could not be confirmed nor refuted by literature. Similarly, little is known about ball skills of children involved in martial arts which probably makes sense since combat sports have little to do with ball skills. Characteristics that do play an important role in martial arts are: flexibility, explosive strength, balance, agility and motor coordination [37]. The latter one does not emerge as distinguishing feature within this study. On the contrary, the children involved in martial arts performed worse on one of the gross motor coordination tests (moving sideways) compared to the rest of the children. Regarding ball skills, it is remarkable that the ball sport players do not outperform the rest of the children, as one would expect considering that ball skills are central in ball sports. This however, does not say that much about the profile of 9 to 11 year old ball sport players but it does unveil a weakness about this specific test for this particular population. With scores between 8.00 and 9.02 (number of correct dribbles with a maximum of 10) (see Table 3), it is likely that the test was to easy for 9 to 11 year old children, which resulted in a ceiling effect, and makes it difficult to find a difference between ball sport players and non-ball sport players.

In the current study, 9 to 11 year old children did not present sport-specific physical characteristics, which could be explained by several reasons. First, the amount of hours spent in a sport may have influenced the physical profile of the children. Sport-specific characteristics are partly the result of what Ericsson [22] called the 10.000 hours rule. Hours and hours of deliberate practice are needed to develop expert performance. In contrast to elites, adolescent athletes who often dedicated years and years of training to their sport, the children within this study (9 to 11 years old) have not spent enough time yet within their sport to demonstrate sport specific characteristics. Adolescent athletes from different types of sports on the other hand, can clearly be distinguished based on their physical profile [18, 19], even when discriminating between sports within the same category. Pion and colleagues [37] found a 100% correct classification when discriminating between three different martial arts sports (judo, karate and tae kwon do) in highly trained U18 male athletes. The assumption that a more extended training history leads to more pronounced sport specific characteristics is supported by the results of the discriminant analyses. Indeed, the current study showed that in 85.2% of the cases, the 81 high active children who spend 5 or more hours per week in their sport were correctly assigned to their proper sport based on their anthropometric, physical fitness and motor coordination profile. In contrast, when considering low active children who spend not more than 1 hour per week, less than half of the children (48.4%) were correctly allocated. Second, it is possible that 9 to 11 year old children do not take into account their physical characteristics when choosing a type of sport. A review on children’s motives for sports participation pointed out the influence of five motivational factors including perception of competence, fun and enjoyment, parents, learning new skills, and friends and peers [38]. Fun and enjoyment is known to be one of the most important motives for children to participate in a sport [39–42]. It is possible that children do not choose a sport that matches their physical qualities in the age range from 9 to 11 but they make that choice based on how much they enjoy the sport.

Regarding talent identification and development, these two viewpoints on exhibiting sport specific characteristics at a young age, can be associated with the nature versus nurture debate, one of the most discussed subjects within this area [43–45]. Nature refers to the innate ability to excel within a sport while nurture means developing skills through an extended amount of high quality training [43]. On the one hand, the difference in sport specific profiles between children who have benefited from a different amount of training hours as found within this study, can be associated with the concept of nurture. The more hours per week a child spends within the sport, the closer it gets to the 10.000 hours which results in exhibiting more pronounced sport specific characteristics. Moreover, an extended training history is not only associated with more pronounced sport specific characteristics; it is also related to better physical fitness and motor coordination qualities. Indeed, results indicated that the children with a better physical fitness and motor coordination profile spend more hours per week in their sport compared to the children who are not quite as strong physically and coordinative. This is supported by a study of Fransen and colleagues [10] who found a positive effect of the amount of training hours per week on the level of physical fitness and motor coordination in 10 to 12 year old boys. Boys who spent few hours per week (<4 hours) in their sport showed poorer motor coordination, flexibility and jumping capabilities compared to boys who spent many hours per week (>4 hours). At the other hand, the assumption that 9 to 11 year old children may not consider their personal characteristics when choosing a sport means that the advantage of an innate ability (nature) goes to waste. To optimize the process of talent identification, children should be supported in choosing a sport that matches their personal characteristics.

Both a genetic potential and optimal environmental factors are favorable to attain a high level of sports performance. However, until now it is not clear whether the nature-nurture debate applies to a broader level of sports participation. The current study elucidated that when children spend a sufficient amount of hours in a sport, they exhibit some sport specific characteristics. It however remains unknown to what degree the children in this particular population chose a sport that matches their personal characteristics. It is possible that the children chose a sport for a different reason (e.g. environmental factors like parental influence) and they exhibit a sport specific profile as a result of many training hours. Meanwhile, there might be another sport that fits better with their anthropometric, physical fitness and motor coordination profile. Future studies should investigate (1) to what extent children need to choose a sport that matches their personal characteristics and (2) whether this well considered choice is better than a choice based on environmental factors like parental influence to protect them from early dropout. In addition, it should be investigated (3) to what degree environmental factors like training volume have an influence on the match between the child and the sport. Furthermore, assuming that a match between the child and the sport is preferable, the question remains whether the elite sport specific profiles apply for 9 to 11 year old children.

One of the strengths of the present study is the large sample size, which made it possible to explore a large number of sports. In addition, unlike many other studies, the focus was on the anthropometric, physical fitness and motor coordination characteristics of children participating in a wide range of sports regardless of their level of sports participation. Despite the large sample size, some sports were not well represented. Therefore, the authors chose to combine sports based on common characteristics. From the viewpoint of talent identification and development it is favorable to focus on an individual sport, rather than on groups of sports.

## References

[1] Barnett LM, Van Beurden E, Morgan PJ, Brooks LO, Beard JR. Childhood Motor Skill Proficiency as a Predictor of Adolescent Physical Activity. J Adolescent Health. 2009;44(3):252–259. 10.1016/j.jadohealth.2008.07.004 19237111

[2] Bauman AE. Updating the evidence that physical activity is good for health: an epidemiological review 2000–2003. J Sci Med Sport. 2004;7(1 Suppl):6–19. 15214597 10.1016/s1440-2440(04)80273-1

[3] Fransen J, Deprez D, Pion J, Tallir IB, D'Hondt E, Vaeyens R, et al Changes in Physical Fitness and Sports Participation Among Children With Different Levels of Motor Competence: A 2-Year Longitudinal Study. Pediatr Exerc Sci. 2014;26(1):11–21. 10.1123/pes.2013-0005 24018944

[4] Gentier I, D'Hondt E, Shultz S, Deforche B, Augustijn M, Hoorne S, et al Fine and gross motor skills differ between healthy-weight and obese children. Res Dev Disabil. 2013;34(11):4043–4051. 10.1016/j.ridd.2013.08.040 24036485

[5] Ortega FB, Ruiz JR, Castillo MJ, Sjostrom M. Physical fitness in childhood and adolescence: a powerful marker of health. Int J Obesity. 2008;32(1):1–11. 10.1038/sj.ijo.0803774 18043605

[6] Sallis JF, Patrick K. Physical activity guidelines for adolescents: consensus statement. Pediatr Exerc Sci. 1994;6(4):302–314.

[7] Fisher A, Reilly JJ, Kelly LA, Montgomery C, Williamson A, Paton JY, et al Fundamental movement skills and habitual physical activity in young children. Med Sci Sport Exer. 2005;37(4):684–688. 15809570 10.1249/01.mss.0000159138.48107.7d

[8] Hands B. Changes in motor skill and fitness measures among children with high and low motor competence: A five-year longitudinal study. J Sci Med Sport. 2008;11(2):155–162. 17567536 10.1016/j.jsams.2007.02.012

[9] Okely AD, Booth ML, Patterson JW. Relationship of physical activity to fundamental movement skills among adolescents. Med Sci Sport Exer. 2001;33(11):1899–1904. 11689741 10.1097/00005768-200111000-00015

[10] Fransen J, Pion J, Vandendriessche J, Vandorpe B, Vaeyens R, Lenoir M, et al Differences in physical fitness and gross motor coordination in boys aged 6–12 years specializing in one versus sampling more than one sport. J Sport Sci. 2012;30(4):379–386. 10.1080/02640414.2011.642808 22214429

[11] Bressel E, Yonker JC, Kras J, Heath EM. Comparison of static and dynamic balance in female collegiate soccer, basketball, and gymnastics athletes. J Athl Training. 2007;42(1):42–46. 17597942 PMC1896078

[12] Duncan MJ, Woodfield L, al-Nakeeb Y. Anthropometric and physiological characteristics of junior elite volleyball players. Brit J Sport Med. 2006;40(7):649–951. 16799112 10.1136/bjsm.2005.021998 PMC2564319

[13] Elferink-Gemser MT, Visscher C, Lemmink KAPM, Mulder T. Multidimensional performance characteristics and standard of performance in talented youth field hockey players: A longitudinal study. J Sport Sci. 2007;25(4):481–489. 17365535 10.1080/02640410600719945

[14] Kirkpatrick J, Comfort P. Strength, Power, and Speed Qualities in English Junior Elite Rugby League Players. J Strength Cond Res. 2013;27(9):2414–2419. 10.1519/JSC.0b013e3182804a6d 23254542

[15] Reilly T, Bangsbo J, Franks A. Anthropometric and physiological predispositions for elite soccer. J Sport Sci. 2000;18(9):669–683. 11043893 10.1080/02640410050120050

[16] Russell M, Tooley E. Anthropometric and performance characteristics of young male soccer players competing in the UK. Serbian journal of sports sciences. 2011;5(4):155–162.

[17] Vandorpe B, Vandendriessche J, Vaeyens R, Pion J, Lefevre J, Philippaerts R, et al Factors Discriminating Gymnasts by Competitive Level. Int J Sports Med. 2011;32(8):591–597. 10.1055/s-0031-1275300 21563024

[18] Leone M, Lariviere G, Comtois AS. Discriminant analysis of anthropometric and biomotor variables among elite adolescent female athletes in four sports. J Sport Sci. 2002;20(6):443–449. 12137174 10.1080/02640410252925116

[19] Pion J, Segers V, Fransen J, Debuyck G, Deprez D, Haerens L, et al Generic anthropometric and performance characteristics among elite adolescent boys in nine different sports. European journal of sport science. 2014:1–10. 25143133 10.1080/17461391.2014.944875

[20] Côté J, Hancock DJ. Evidence-based policies for youth sport programmes International Journal of Sport Policy and Politics 2014;6(3):In press.

[21] Côté J, Lidor R, Hackfort D. ISSP Position Stand: To sample or to specialize? Seven postulates about youth sport activities that lead to continued participation and elite performance. International Journal of Sport and Exercise Psychology. 2009;9:7–17.

[22] Ericsson KA, Krampe RT, Teschromer C. The Role of Deliberate Practice in the Acquisition of Expert Performance. Psychological Review. 1993;100(3):363–406.

[23] Vandorpe B, Vandendriessche J, Vaeyens R, Pion J, Matthys S, Lefevre J, et al Relationship between sports participation and the level of motor coordination in childhood: A longitudinal approach. J Sci Med Sport. 2012;15(3):220–225. 10.1016/j.jsams.2011.09.006 22047725

[24] Matthys SP, Vaeyens R, Fransen J, Deprez D, Pion J, Vandendriessche J, et al A longitudinal study of multidimensional performance characteristics related to physical capacities in youth handball. J Sports Sci. 2013;31(3):325–334. 10.1080/02640414.2012.733819 23078540

[25] Vandendriessche JB, Vandorpe BFR, Vaeyens R, Malina RM, Lefevre J, Lenoir M, et al Variation in Sport Participation, Fitness and Motor Coordination With Socioeconomic Status Among Flemish Children. Pediatr Exerc Sci. 2012;24(1):113–128. 22433257 10.1123/pes.24.1.113

[26] Europe Co. Testing physical fitness: Eurofit Experimental Battery—Provisional Handbook. Strasbourg: Council of Europe; 1983.

[27] Vrijkotte S, De Vries S, Jongert T. Fitheidstesten voor de jeugd. Leiden: TNO Kwaliteit van Leven; 2007.

[28] Fetz F, Kornexl E. Sportmotorische Tests. Berlin: Bartels & Wernitz; 1993.

[29] Johnson BL, Nelson JK. Practical measurements for evaluation in physical education. 4th ed. Edina: Minneapolis: Burgess Publishing Company; 1986.

[30] Markovic G, Dizdar D, Jukic I, Cardinale M. Reliability and factorial validity of squat and counter movement jump tests. J Strength Cond Res. 2004;18(3):551–555. 15320660 10.1519/1533-4287(2004)18<551:RAFVOS>2.0.CO;2

[31] Cometti G, Cometti D. La pliométrie: Méthodes, entraînements et exercices. Paris: Chiron; 2007.

[32] Bruininks RH, Bruininks BD. BOT-2: Bruininks-Oseretsky Tests of Motor Proficiency. Minneapolis: AGS Publishing; 2006.

[33] Kiphard EJ, Schilling F. Körperkoordinationstest für Kinder 2 Überarbeitete und ergänzte Auflage. Weinheim: Beltz: Test GmbH 2007.

[34] Philippaerts RM, Matton L, Wijndaele K, Balduck AL, De Bourdeaudhuij I, Lefevre J. Validity of a physical activity computer questionnaire in 12-to 18-year-old boys and girls. Int J Sports Med. 2006;27(2):131–136. 16475059 10.1055/s-2005-837619

[35] Bencke J, Damsgaard R, Saekmose A, Jorgensen P, Jorgensen K, Klausen K. Anaerobic power and muscle strength characteristics of 11 years old elite and non-elite boys and girls from gymnastics, team handball, tennis and swimming. Scand J Med Sci Spor. 2002;12(3):171–178. 10.1034/j.1600-0838.2002.01128.x 12135450

[36] Carrick FR, Oggero E, Pagnacco G, Brock JB, Arikan T. Posturographic testing and motor learning predictability in gymnasts. Disabil Rehabil. 2007;29(24):1881–9. 17852265 10.1080/09638280601141335

[37] Pion JF J.; Lenoir M.; Segers V. The value of non-sport-specific characteristics for talent orientation in young male judo, karate and taekwondo athletes. Arch Budo. 2014;10:147–154.

[38] Cope EJ, Bailey R, Pearce G. Why do children take part in, and remain involved in sport? A literature review and discussion of implications for sports coaches. International Journal of Coaching Science. 2013;7(1):55–74.

[39] Chalip L, Green BL. Establishing and maintaining a modified youth sport program: Lessons from Hotellings location game. Sociology of Sport Journal 1998;15(4):326–42.

[40] Green BC. Building sport programs to optimize athlete recruitment, retention, and transition: Toward a normative theory of sport development. J Sport Manage. 2005;19(3):233–253.

[41] Wankel LM, Kreisel PSJ. Factors Underlying Enjoyment of Youth Sports—Sport and Age Group Comparisons. J Sport Psychol. 1985;7(1):51–64.

[42] Weiss MR, Amorose AJ. Motivational orientations and sport behaviour In: Horn TS, editor. Advances in Sport Psychology. 3 ed. Champaign, IL: Human Kinetics; 2008 p. 115–155.

[43] Davids K, Baker J. Genes, environment and sport performance: why the nature-nurture dualism is no longer relevant. Sports medicine. 2007;37(11):961–80. 17953467 10.2165/00007256-200737110-00004

[44] Durand-Bush N, Salmela JH. The Development of Talent in Sport In: Singer RN, H H. A.; Janelle C. M., editor. Handbook of Sport Psychology 2ed. Canada: John Wiley & Sons; 2001 p. 269–289.

[45] Tucker R, Collins M. What makes champions? A review of the relative contribution of genes and training to sporting success. Br J Sports Med. 2012;46(8):555–561. 10.1136/bjsports-2011-090548 22535537

